# Polychlorinated Biphenyls and Pulmonary Hypertension

**DOI:** 10.3390/ijerph19084705

**Published:** 2022-04-13

**Authors:** Hamza Assaggaf, Changwon Yoo, Roberto G. Lucchini, Steven M. Black, Munerah Hamed, Faisal Minshawi, Quentin Felty

**Affiliations:** 1 Department of Laboratory Medicine, Faculty of Applied Medical Sciences, Umm Al-Qura University, Makkah 21955, Saudi Arabia; hmsaggaf@uqu.edu.sa (H.A.); fominshawi@uqu.edu.sa (F.M.); 2 Department of Biostatistics, Florida International University, Miami, FL 33199, USA; cyoo@fiu.edu; 3 Department of Environmental Health Sciences, Florida International University, Miami, FL 33199, USA; rlucchin@fiu.edu; 4 Department of Medical Surgical Specialties, Radiological Sciences and Public Health, University of Brescia, 25123 Brescia, Italy; 5 FIU-Center for Translational Science, Port St. Lucie, FL 34987, USA; stblack@fiu.edu; 6 Department of Pathology, Faculty of Medicine, Umm Al-Qura University, Makkah 21955, Saudi Arabia; mhhamed@uqu.edu.sa

**Keywords:** PCBs, NHANES, pulmonary arterial hypertension

## Abstract

Polychlorinated biphenyls (PCBs) are persistent environmental pollutants that were banned because of their potential carcinogenicity. Population studies have shown that PCBs are associated with lung toxicity and hypertension. The objective of this study was to evaluate whether higher exposure to PCB congeners is associated with the risk of pulmonary hypertension. Serum levels of PCBs in 284 subjects with combined risk factors for pulmonary arterial hypertension (PAH) were compared to 4210 subjects with no risk for PAH using the National Health and Nutrition Examination Survey (NHANES) from 1999 to 2004. The major findings from this study include significantly higher PCB levels in PAH subjects compared to non-PAH subjects; for example, the geometric mean (GM) of PCB74 was 15.91 (ng/g) (14.45–17.53) vs. 11.48 (ng/g) (10.84–12.16), respectively. Serum levels of PCB congeners showed an increasing trend in the age group 20–59 years as PCB180 GM was 19.45 (ng/g) in PAH vs. 12.75 (ng/g) in the control. A higher body burden of PCB153 followed by PCB138, PCB180, and PCB118 was observed. Estimated age, race, BMI, and gender-adjusted ORs for PCB congener levels in subjects with the combined risk factors for PAH compared to controls was significant; for example, PCB99 (OR: 1.5 (CI: 1.49–1.50). In summary, these findings indicate that exposure, as well as body burden estimated based on lipid adjustment of PCBs, were higher in people with risk factors for PAH, and PCB congeners accumulated with age. These findings should be interpreted with caution because of the use of cross-sectional self-reported data and a small sample size of subjects with combined risk factors for pulmonary arterial hypertension. Nonetheless, our finding emphasizes a need for a comprehensive environmental molecular epidemiologic study to determine the potential role of environmental exposures to PCBs in the development of pulmonary arterial hypertension.

## 1. Introduction

Polychlorinated biphenyls (PCBs) are anthropogenic compounds that were used in electrical equipment, hydraulic machinery, and as additives in caulking compounds, paints, adhesives, flame retardants, and plasticizers. Although PCBs have been banned due to their potential carcinogenicity, they still pose serious risks to human health because of their long-term chemical stability and widespread contamination of the environment. Inhalation of airborne PCBs is now considered an important route of exposure in the general population. Studies showed that airborne exposure to PCBs from building materials occurs in schools [1]. In vivo studies confirmed that inhalational exposure to indoor school air mixtures of PCBs induced oxidative stress in the lungs [2]. PCB-induced vascular toxicity through reactive oxygen species (ROS) initiated inflammation leads to endothelial injury. We showed that PCB153 congener-induced oxidative stress in endothelial cells increased the angiogenic phenotype, including tube branching, sprouting, spheroid growth, and proliferation [3]. Since PCB-induced oxidative stress supports the growth of vascular endothelial cells, we postulate that exposure to PCBs may be associated with proliferative and obliterative vascular disease.

Pulmonary arterial hypertension (PAH) is a vascular disease that affects pulmonary arteries. Obliterative vascular lesions consisting of hyperproliferative smooth muscle and endothelial cells cause increased mean pulmonary arterial pressure [4]. This results in symptoms such as fatigue and shortness of breath, but the major cause of increased morbidity and mortality is right ventricular dysfunction. PAH patients with end-stage occlusive vasculopathy have a poor prognosis for survival of 5–7 years even after treatment [5]. Multiple factors drive the pathogenesis of PAH, including sex, genetic predisposition, epigenetics, inflammation, and metabolism. Women are up to four times as likely to present with PAH than men, but male patients are consistently shown to have more severe PAH and poor survival [6,7]. Sex differences were reported in the France national registry, with females at higher risk of PAH than males (OR 1.9; *p* < 0.035) [8]. Moreover, the REVEAL study reported the highest female to male ratio of 4:1 in idiopathic PAH [9]. The sex differences may be explained partly by estrogen and estrogen metabolites. Alterations in estrogen metabolism via CYP1B1 modify the risk of familial PAH [10]. Additionally, oral contraceptives and hormone replacement therapies were shown to be associated with severity of PAH and formation of obliterative lesions in idiopathic PAH [11,12]. However, the most widely studied factor in PAH is genetic predisposition from mutations in genes in familial and sporadic PAH. Mutations in the BMPR2 gene are a common cause of PAH observed in approximately 80% of familial PAH and 20% of sporadic cases [13]. Other genes such as ALK1, ENG, SMAD9, CAV1, KCNK3, and SOX17 have also been associated with PAH [14,15,16,17,18,19]. Epigenetic mediators of PAH include the methyltransferase DNMT3B which is upregulated in PAH patients [20]. Inflammatory mediators such as cytokine IL-1β and IL-6 have been correlated with worse outcomes of PAH [21,22]. Proliferative cells in PAH show a metabolic shift to glycolysis. The accumulation of hypoxia-inducible factor (HIF)-1α promotes the shift to glycolysis and away from oxidative glucose metabolism in PAH [23]. Metabolic derangements in arginine metabolism also cause vasoconstrictive phenotype of PAH [24].

Other risk factors reported to be associated with PAH include hypertension, abnormal lung function, diabetes, uric acid level, age, insulin status, obesity, thyroid problems, and female sex hormones. Some of these same risk factors contribute to metabolic syndrome. A high prevalence of metabolic syndrome reported in PAH patients suggests that it contributes to the progression of pulmonary hypertension [25]. Evidence of the previously mentioned risk factors for PAH are from the following studies. Insulin resistance (IR) was reported to be associated with PAH and poor survival [26]. The Registry to Evaluate Early and Long-term PAH Disease Management (REVEAL Registry) showed a significant association between obesity (BMI > 30, *p*-value = 0.004) and PAH when compared to normal and underweight patients [27]. Zhang et al. (2013) showed that serum uric acid levels in PAH patients (405 ± 130 μmol/L) were higher than in control subjects (344 ± 96 μmol/L; *p* < 0.05) [28]. Reference values were 360 μmol/L and 420 μmol/L for females and males, respectively. Older age was reported to be a risk factor for PAH compared with younger patients. Schachna et al. (2003) showed that the elderly >60 years old had a two times higher risk of PAH than patients <60 years old (OR, 2.30; 95% CI, 1.32 to 3.99) [29]. Many studies showed a higher prevalence of thyroid problems and risk of PAH. Li et al. (2007) reported that the risk of PAH was two times higher in individuals with thyroid disease (OR, 2.53; 95% confidence interval, 1.55 to 4.08; *p* < 0.001) [30]. The prevalence of PAH was shown to be increased by up to 50% in type 2 diabetes [31]. Based on these studies, we identified a population with a combination of risk factors for PAH (hypertension, diabetes, thyroid problems, abnormal uric acid level, and insulin resistance) in the National Health and Nutrition Examination Survey (NHANES) to determine whether or not they had a higher level of exposure to PCBs. We aim to (i) describe the mean concentration of individual PCB congeners as well as the sum of dioxin- and non-dioxin-like PCBs in the population with combined risk factors for PAH compared to subjects with no risk factors; and (ii) compare different limit of detection levels of PCBs to evaluate whether higher concentrations of PCBs are associated with these combined risk factors for PAH.

## 2. Methods

### 2.1. Study Population

NHANES provides biomonitoring data for PCBs in the civilian noninstitutionalized U.S. population. The study population came from NHANES cycles 1999–2004 provided by the Centers for Disease Control and Prevention. All of the data on exposure and outcome variables from NHANES cycles 1999–2004 were analyzed retrospectively. NHANES is a program developed by the National Center for Health Statistics to assess the nutritional and health status of children and adults in the United States. The NHANES health survey of the US population is conducted in two-year cycles. Cross-sectional population-based datasets are generated from medical questionnaires and include demographic information, diet and nutrition data, medical and health conditions, and collected biological samples (blood and urine), which are obtained through mobile examinations center (MEC). The National Center for Health Statistics Institutional Review Board approved all NHANES protocols, and all participants provided written, informed consent, and child permission before any data or sample collection. Blood serum and urine samples were collected from 2500 participants in each 2-year cycle. Exposure assessments of environmental chemicals were carried out by the Division of Laboratory Sciences, National Center for Environmental Health, and CDC’s Environmental Health Laboratory. Serum blood samples were measured per whole weight or per gram of total lipid to reflect the number of PCBs stored in body fat. Both urine and blood serum samples were stored at 4 °C or froze at −20 °C before shipping to the Division of Laboratory Sciences, National Center for Environmental Health, CDC’s Environmental Health Laboratory [32]. The one-third sampling procedure was used to measure PCB levels in blood serum samples of participants ≥12 years old in the 1999–2000 and 2003–2004 survey cycles. In the 2001–2002 survey cycle, NHANES used age inclusion to be ≥20 years old. PCBs were analytically reported on a lipid-adjusted basis (ng/g). 

### 2.2. Study Design

Risk factors reported to be associated with PAH include systemic hypertension, diabetes, uric acid level, age, insulin status, obesity, thyroid problems, and hormone therapy [11,12,26,27,28,29,30,33]. Based on these studies, we identified a population with a combination of risk factors for PAH (hypertension, diabetes, thyroid problems, abnormal uric acid level, and insulin resistance) in the NHANES. Male subjects with two or more of these risk factors for PAH and female subjects with three or more of these risk factors (including hormone therapy) were identified to have combined risk factors for PAH defined as “subjects with combined risk factors for PAH” shown in Table 1 [34]. Participants were not included if questions for any of these risk factors were not answered or medical lab data were missing from their interview. Using information from the medical health questionnaire, the participants had to provide a response to the combine risk factors for PAH, including “have you ever been told by a doctor or health professional that you have diabetes?” and “Has a doctor or other health professional ever told that you had a thyroid disease?”. Diabetes risk factor was scored 0 if answer was “no”, and scored 1 if answer was “yes”. Thyroid problem risk factor was scored 0 if answer was “no”, and scored 1 if answer was “yes”. Each of these variables was transformed into 0 if no risk factors for PAH and 1 if subjects had combined risk factors for PAH. Using the reproductive health questionnaire, female participants >20 years old had to provide a response to the question “have you ever used female hormones such as estrogen and progesterone?” Female hormone risk factors (female participants only) was scored 0 in the female participant answered “no”, and scored 1 if the female participant answered “yes”. Using medical lab data measurements, uric acid level was available for the included participant, as well as measurements of both (triglycerides and HDL-cholesterol) to calculate insulin status (insulin sensitive or insulin resistance). Uric acid risk factor is a continuous variable, so males and females have different references. Male participants scored 0 if with uric acid level <419 mmol/L, and scored 1 with a uric acid level >420 mmol/L. Female participants scored 0 if with uric acid level <359 mmol/L, and scored 1 if any male participants had a uric acid level >360 mmol/L. Insulin status was calculated by dividing triglycerides level by HDL-cholesterol level. Participants with insulin status <1.99 were categorized as “insulin sensitive (IS)” with a score of 0. If insulin status was >2.00, then participants were considered to be “insulin resistant (IR)” with a score of 1.

### 2.3. Selection of PCBs

The number of PCB congeners varied in different NHANES cycles (1999–2000, 2001–2002, and 2003–2004). In order to avoid bias from low detection limited samples, a total of six PCB congeners that had measurements above 60% of the population samples were used in this study. Based on this inclusion criteria, the PCB congeners were: 2,4,4′,5-tetrachlorobiphenly (PCB74), 2,2′4,4′,5-pentachlorobiphenyl (PCB99), 2,3′,4,4′,5-pentachlorobiphenyl (PCB118), 2,2′,3,4,4′,5-hexachlorobiphenyl (PCB138), 2,2′,4,4′,5,5′-hexachlorobiphenyl (PCB153), and 2,2′,3,4,4,5,5′-heptachlorobiphenyl (PCB180). We also analyzed the sum of dioxin-like PCBs (PCB74 + PCB118) and of non-dioxin-like PCBs (PCB99 + PCB138 + PCB153 + PCB180). By using complex survey NHANES data for merged cycles from 1999 to 2004, we analyzed the serum levels of PCBs associated with subjects with risk factors of PAH compared to controls with no risk factors. 

### 2.4. Sample Weights and Limits of Detection

The sample weight is required when merging NHANES complex survey data to avoid bias from non-response selection. As mentioned earlier, PCB measurements were collected in the one-third selection procedure, so weighting these environmental chemicals is also required. The weight variable was provided in each 2-year cycle from 1999 to 2004 for all variables, and it was created for the environmental chemical based on NHANES analytic guidelines [35]. The detection limit for each of the environmental chemicals was divided into 0 if below the detection limit and 1 if above the detection limit for PCB. However, if the PCB serum level was below the detection limit, it is required to be divided by the square root of 2. CDC improved their procedure and techniques yearly, which resulted in changes in the detection limit of environmental chemicals [32]. Individual PCB congeners have their own LOD because they have different sample volumes. Thus, calculations for each of the environmental chemicals are required based on sample volume for each serum sample to be analytically sensitive. Serum samples of environmental chemicals were calculated per amount of lipid [35]. 

### 2.5. Statistical Analysis

We selected people >20 years old with data for PCBs, medical health, and reproductive health to find the association between PCB levels and combined risk factors for PAH. Because of the non-normal distribution of PCBs measurements, they were log-transformed before the analysis. Moreover, data were weighted using the required weight by the National Center for Health Statistics guidelines to be representative of the whole population. We used stratum and PSA to estimate the variance of the demographic data. We calculated geometric means (GM), geometric standard errors (GSE), and proportions for all PCBs and their association with the combined risk factors for PAH. We used Student’s t-test to compare PCBs levels in different groups. In order to find the associated probability of higher PCB levels with combined risk factors for PAH with each of the PCBs, we used logistic regression models to calculate the odds ratios (ORs) and their 95% confidence intervals (CI). Because of the small number of participants who had combined risk factors for PAH, we used NCHS guidelines to categorize the limit of detection (LOD) into many levels to perform different analyses based on the LOD (51). These LOD include (1) <LOD vs. ≥LOD; (2) <LOD to 50th percentile vs. ≥50th percentile; and (3) <LOD to 50th percentile vs. 50th percentile to 75th percentile vs. ≥75th percentile. Statistical analyses were performed using SPSS software (release 20) for windows and SAS software for windows (release 9.4; SAS Institute Inc, Cary, NC, USA). We used 5% (*p* ≤ 0.05) as the significance level for all analyses.

### 2.6. Non-Identifying Individual-Level Health Data including Demographics, Nutrition, and Other Factors

Non-identifying individual-level health data such as gender, age, body mass index (BMI, kg/m^2^), annual family income, smoking status, alcohol consumption, race, and education level were included in the analysis. Age was categorized into three age groups: 20–59 years, 60–74 years, and ≥75 years. BMI was categorized into three weight groups: >25 kg/m^2^ (normal weight), 25–30 kg/m^2^ (overweight), and ≥30 kg/m^2^ (obese). Annual family income was categorized into the following groups: USD 0–24,999, USD 25,000–USD 54,999, USD 55,000–USD 74,999, and USD ≥ 75,000. Smoking status was categorized into smokers and non-smokers, and alcohol consumption was categorized into consumers and non-consumers. Race was categorized into non-Hispanic White and others. Education level was categorized into three categories: <12th grade, 12th grade, and >12th grade. 

## 3. Results

The National Health and Nutrition Examination Survey (NHANES) provides nationally representative biomonitoring data for PCBs. The population used in this study comes from NHANES year cycles 1999–2000, 2001–2002, and 2003–2004. We selected six PCB congeners measured in serum for these time periods in more than 60% of the samples. The selected PCB congeners were PCB74, PCB99, PCB118, PCB138, PCB153, and PCB180. We also included the sum of dioxin-like PCBs (PCB74+PCB118) and non-dioxin-like PCBs (PCB99+PCB138+PCB153+PCB180). The combined risk factors reported to be associated with PAH include systemic hypertension, diabetes, uric acid level, age, insulin status, obesity, thyroid problems, and female sex hormones [11,12,26,27,28,29,30,33]. Some of these risk factors were also associated with metabolic syndrome. A high prevalence of metabolic syndrome reported in PAH patients suggests that it contributes to the progression of pulmonary hypertension [25]. Male subjects with two or more of the following risk factors for PAH (systemic hypertension, diabetes, thyroid problems, abnormal uric acid level, insulin resistance) and female subjects with three or more of these risk factors (including hormone therapy) were identified to have combined risk factors for PAH defined as “subjects with combined risk factors for PAH” (Table 1). Individuals with no risk factors for PAH were included in the no-risk factor or control group. Descriptive statistics for subjects with combined risk factors for PAH and the control group are shown in Table 2. The majority of study participants with combined risk factors for PAH were male or between 60 and 74 years old compared to participants not having risk factors for PAH. The majority of subjects with combined risk factors for PAH responded “no” to a history of smoking and alcohol use and reported a yearly family income between USD 0 and 54,999.

The geometric mean (GM) for blood levels of PCB congeners was calculated in subjects with the combined risk factors for PAH and controls shown in Table 3. PCB levels were expressed on a lipid-adjusted basis to represent body burden better. The age-adjusted GM for individual PCB congeners in the control group ranged from 5.29 to 27.39 ng/g lipid in the control group and from 6.35 to 35.48 ng/g lipid in subjects with combined risk factors for PAH. Levels of all PCB congeners were significantly higher in age-adjusted subjects with risk factors for PAH when compared to the control age-adjusted group. Among the six PCB congeners, PCB153 had the highest level observed in participants with combined risk factors for PAH with the lowest level from PCB99. Consistent with previous findings that PCBs are associated with obesity [36], we observed the highest number of subjects with combined risk factors for PAH in the elevated BMI group categorized as “obese” (Table 2), suggesting a higher body burden of PCB153, which is responsible for vascular impacts.

PCB levels measured above the limit of detection (LOD) were examined by age group in subjects with the combined risk factors for PAH and controls with no risks shown in Table 4. The overall GM of PCBs found in serum increased with age for each individual PCB congener. PCB153 showed the highest serum levels in subjects with combined risk factors for PAH across age groups: 35.47 ng/g lipid in 20–59 yr, 64.06 ng/g lipid in 60–74 yr, and 84.56 ng/g lipid in ≥75yr old subjects. Levels of PCBs in subjects with combined risk factors for PAH were significantly higher in the 20–59 yr age group.

Age-adjusted PCB levels in the blood were determined by the race of participants, shown in Table 5. The lipid-adjusted levels of all PCB congeners in subjects with risk factors for PAH were almost doubled in subjects in the category “other” when compared to their respective control. Control levels of PCB congeners were significantly higher in non-Hispanic White compared to the category “other” for PCBs 74, 138, 153, and 180. Levels of PCBs in subjects with combined risk factors for PAH were significantly higher in the race category of “other” compared to non-Hispanic Whites for PCB 99, 138, 153, and 180.

Concentrations of PCB congeners that elicit dioxin-like toxicity were examined as the sum of dioxin-like PCB congeners (PCB74+PCB118). We calculated arithmetic means of serum levels of dioxin-like PCBs shown in Table 6. Overall the dioxin-like PCB concentration in serum was significantly higher in participants with the combined risk factors for PAH (3.36 ng/g) compared to the control group (2.81 ng/g) (Table 6).

The sum of non-dioxin-like PCBs (99 + 138 + 153 + 180) was also determined in the participants with combined risk factors of PAH (Table 7). Participants with combined risk factors for PAH reported a significantly higher mean of non-dioxin-like PCBs compared to control (4.88 ng/g vs. 4.40 ng/g) (Table 7). We also evaluated the association of covariates with combined risk factors for PAH and their association with the mean concentration of non-dioxin-like PCBs, including gender, race, age, BMI, annual family income, education level, smoking, and alcohol use. Most covariates reported a higher mean of non-dioxin-like PCBs in subjects with combined risk factors for PAH compared to control. 

Geometric means using the LOD of individual PCB congeners were analyzed in the study population to evaluate their association with the combined risk factors of PAH. We reported the GMs of PCBs with 95% CI for each LOD level and observed that all PCB GMs ≥LOD were higher in subjects with combined risk factors for PAH compared to controls (Table 8). GMs ≥ LOD for individual PCB congeners in subjects with combined risk factors for PAH were 15.91 ng/ng (PCB74), 10.99 ng/ng (PCB99), 21.47 ng/ng (PCB118), 42.17 ng/ng (PCB138), 58.30 ng/ng (PCB153), and 45.49 ng/ng (PCB180) (Table 8). Control GMs ≥ LOD for individual PCB congeners were 11.48 ng/ng (PCB74), 8.36 ng/ng (PCB99), 15.29 ng/ng (PCB118), 33.58 ng/ng (PCB138), 46.79 ng/ng (PCB153), and 37.90 ng/ng (PCB180) (Table 8). 

Because of the small number of people with combined risk factors for PAH, we analyzed GMs of individual PCB congeners with LOD < 50th percentile and ≥50th percentile (Table 9). 

Study participants with combined risk factors for PAH reported GMs ≥ 50th percentile LOD of 17.73 ng/ng (PCB74), 12.59 ng/ng (PCB99), 24.21 ng/ng (PCB118), 48.84 ng/ng (PCB138), 69.33 ng/ng (PCB153), and 53.89 ng/ng (PCB180) (Table 9). In controls with no risk factors for PAH, GMs for PCBs ≥ 50th percentile LOD were 15.17 ng/ng (PCB74), 10.53 ng/ng (PCB99), 19.01 ng/ng (PCB118), 43.59 ng/ng (PCB138), 63.33 ng/ng (PCB153), and 49.33 ng/ng for PCB180 (Table 9). Overall, no significant difference was reported between GM PCB concentrations of people with combined risk factors for PAH and the control group with no risk (Table 9). Therefore, we performed another analysis stratifying the LODs for PCBs using <LOD to 50th percentile, LOD 50th percentile to 75th percentile, and LOD ≥ 75th percentile (Table 10). A higher number of subjects with combined risk factors for PAH were reported among LOD ≥ 75th percentile compared to <LOD to 50th percentile and 50th percentile to 75th percentile (Table 10). Levels of PCBs were higher in subjects with combined risk factors for PAH compared to the control group for all PCB congeners except PCB99 at the LOD ≥ 75th percentile (Table 10).

We calculated the odds ratio (OR) and 95% confidence intervals to estimate the probability of higher PCB congener levels of more than 50% LOD in participants with the combined risk factors of PAH (Table 11). After adjusting for age, race, BMI, and gender, we observed that subjects with combined risk factors for PAH had a greater chance of higher serum PCB concentrations when compared to the control. The highest OR was found in PCB74 with [OR of 2.31; 95% CI: 2.30–2.32] (Table 11).

We also calculated the OR and 95% confidence intervals to estimate the probability of higher PCB congener levels with the combined risk factors of PAH with LOD in the 50th to 75th percentile and ≥75th percentile (Table 12). In age, race, BMI, and gender-adjusted OR, we observed that subjects with combined risk factors for PAH had a greater chance of higher serum PCB concentrations for all congeners at LOD 50–75%. The highest OR was found in PCB74 LOD levels ≥ 75% with [OR of 2.65; 95% CI: 2.63–2.66]

We calculated the estimated OR and 95% confidence intervals to estimate the probability of higher dioxin-like or non-dioxin-like PCBs levels with the combined risk factors of PAH compared to controls (Table 13). In the age, race, BMI, and gender-adjusted OR, we observed that non-dioxin-like PCBs at LOD levels ≥50th percentile and ≥75th percentile were not associated with participants with combined risk factors for PAH (Table 13). Dioxin-like PCBs showed a greater probability to be in subjects with combined PAH risk at LOD levels ≥ 50% [OR of 1.73; 95% CI: 1.72–1.74] and ≥75% [OR of 1.75; 95% CI: 1.75–1.76].

## 4. Discussion

This is the first cross-sectional study to evaluate whether higher exposure to PCB congeners is associated with the risk of PAH. The major findings from this study include levels of PCB congeners were significantly higher in subjects with risk factors for PAH. Among the six PCB congeners, PCB153 had the highest level observed in participants with risk factors for PAH. Serum levels of PCB congeners showed an increasing trend with age. A higher body burden of PCB153 followed by PCB138, PCB180, and PCB118 was observed. Estimated ORs for PCB congener levels in subjects with the combined risk factors for PAH compared to control were significant. In summary, these findings indicate that exposure, as well as body burden of PCBs, was higher in people with risk factors for PAH and PCB congeners tend to accumulate with age. 

Epidemiological studies showed that chronic exposure to PCBs is associated with lung toxicity and hypertension [37,38]. Moreover, occupational exposure to chlorinated solvents was correlated with an increased risk of pulmonary hypertension [39]. High levels of PCBs were reported in human lung tissue [40]. Inhalation exposure to vapor-phase PCBs was demonstrated to be even more important than ingestion under some circumstances [41]. PCBs damage to the vasculature was associated with an increased risk for cardiovascular disease in several population studies [42,43,44,45,46,47]. PCB-induced vascular toxicity through ROS-initiated inflammation may lead to endothelial injury and pulmonary vessel remodeling. We have previously shown that PCB-induced ROS increases angiogenesis and the growth of vascular spheroids [3]. The growth of neointimal lesions in patients with PAH eventually obliterates the vascular lumen and contributes to increased pulmonary arterial pressure. We reported that PCB153 [1 ng/mL] at a level found in human serum [0.60–1.63 ng/mL] altered the angiogenic phenotype of vascular endothelial cells. PCB153 effects are even more pronounced than the hormone estrogen with respect to vasculosphere formation and vasculogenesis [48]. Furthermore, we were able to inhibit PCB-induced endothelial tube branching by silencing the focal adhesion kinase Pyk2 [3]. Pyk2 was shown to contribute to hypoxia-induced PAH in the animal model [49], but it is not known whether PCB-induced vascular toxicity via Pyk2 may also contribute to PAH. Therefore, our study findings show the association of elevated PCBs in subjects with risk factors for PAH may be linked to mechanisms involved with PCB-induced vascular toxicity.

Our analysis of serum blood PCB levels in the 1999–2004 NHANES survey cycles showed that exposure, as well as body burden of PCBs, was higher in people with risk factors for PAH, and PCB congeners tend to accumulate with age. These findings should be interpreted with caution because of the use of cross-sectional self-reported data and a small sample size of subjects with combined risk factors for PAH. We selected subjects based on combined risk factors for PAH because there was no reported information for PAH in the NHANES survey. This increases the risk of misclassification of people with combined risk factors for PAH. However, many of the same risk factors used in our study to determine subjects with combined risk factors for PAH are also risk factors for metabolic syndrome; and our results are partly supported by another study showing a significant association between PCBs and metabolic syndrome in non-diabetic adults from NHANES survey 1999–2002 [50]. Since there is a high prevalence of metabolic syndrome in PAH patients, further comprehensive research is needed to determine the potential role of PCB exposures and molecular mechanisms of PCB-induced vascular toxicity in pulmonary hypertension.

## Figures and Tables

**Table 1:** Selection criteria identifying subjects with combined risk factors for PAH.

Risk Factors	NHANES Questionaire	Subjects with Risk Factors for PAH **	Subjects with no Risk Factors for PAH
Systemic Hypertension	Yes/No	Yes = 1	No = 0 (Zero)
Diabetes	Yes/No	Yes = 1	No = 0 (Zero)
Thyroid Problems	Yes/No	Yes = 1	No = 0 (Zero)
Uric Acid Level			
Male	Normal (<419 mmol/L) Abnormal (>420 mmol/L)	Abnormal = 1	Normal = 0
Female	Normal (<359 mmol/L) Abnormal (>360 mmol/L)	Abnormal = 1	Normal = 0
Insulin Status *	Normal (<1.99, Insulin Sensitive (IS)) Abnormal (>2.00, Insulin Resistance (IR))	IR = 1	IS = 0
Female Hormone (Only for Female Participants).	Yes/No	Yes = 1	No = 0 (Zero)

* Insulin status: Calculated as triglycerides level/HDL–cholesterol level. If <1.99 “insulin sensitive (IS)” or if >2.00 “insulin resistance (IR)”. ** subjects with risk factors for PAH (sum of risk score, this means maximum (5 for males) and (6 for females since they have 1 extra criterion (using hormones). Thus, participants were selected in the group with risk factors of PAH if the total score was >2 in males or >3 in females.

**Table 2:** Descriptive statistics for individuals with combined risk factors for PAH and no risk with selected covariates among the population ≥20 years of age, NHANES 1999–2004.

Variable	Subjects with Combined Risk Factors for PAH *n* (%)	Subjects with No Risk Factors for PAH *n* (%)
**Total Population (*n*,%)**	284 (5.08%)	4210 (94.92%)
**Gender**		
Male	187 (3.16%)	1930 (45.02%)
Female	97 (1.92%)	2280 (49.9%)
**Race**		
Non-Hispanic White	161 (4.07%)	2133 (67.78%)
Other	123 (1.01%)	2077 (27.14%)
**Age (years)**		
20–59	48 (2.38%)	2851 (75.17%)
60–74	130 (1.79%)	854 (13.17%)
≥75	70 (0.91%)	505 (6.58%)
**BMI (kg/m^2^)**		
Normal Weight	36 (0.71%)	1062 (34.88%)
Overweight	91 (1.99%)	1112 (32.76%)
Obese	140 (3.78%)	913 (25.89%)
**Income (yearly family income)**		
USD 0–24,999	140 (1.91%)	1633 (28.49%)
USD 25,000–54,999	73 (1.53%)	1216 (28.30%)
USD 55,000–74,999	15 (0.53%)	385 (10.96%)
≥75,000	56 (1.11%)	976 (27.16%)
**Education**		
<12th Grade	112 (1.42%)	1342 (18.97%)
12th Grade	72 (1.54%)	963 (23.59%)
≥12th Grade	100 (2.12%)	1898 (52.34%)
**Smoking**		
Yes	47 (2.15%)	693 (46.02%)
No	139 (6.45%)	824 (45.38%)
**Alcohol use**		
Yes	0	20 (0.77%)
No	282 (6.59%)	3140 (92.63%)

Estimated percent distribution after applying NHANES sampling weights.

**Table 3:** Geometric mean of PCB levels by combined risk factors for PAH and without risk among population ≥20 years of age with PCB concentration above the LOD, NHANES 1999–2004.

		Geometric Mean ^2^ (ng/g) (GSE)			
Analyte ^1^	Controls/Subjects with Combined Risk Factors for PAH (*n*)	Control Unadjusted Levels (ng/g) of PCBs	Unadjusted Levels of PCBs in Subjects with Risk Factors for PAH	Control Age-Adjusted Levels (ng/g) of PCBs ^3^	Age-Adjusted Levels of PCBs in Subjects with Risk Factors for PAH ^3^
**PCB74**	2904/272	7.49 (0.25)	12.76 (0.70) ^a^	6.08 (0.20)	8.62 (0.72) ^a^
**PCB99**	2874/267	6.03 (0.15)	8.43 (0.33) ^a^	5.29 (0.14)	6.35 (0.37) ^a^
**PCB118**	2904/272	8.85 (0.26)	15.43 (0.84) ^a^	7.25 (0.20)	10.11 (0.85) ^a^
**PCB138**	2908/272	22.54 (0.50)	34.35 (1.59) ^a^	19.13 (0.44)	23.56 (1.65) ^a^
**PCB153**	2908/273	32.64 (0.73)	51.13 (2.42) ^a^	27.39 (0.63)	35.48 (2.55) ^a^
**PCB180**	2905/273	16.79 (0.59)	33.86 (2.39) ^a^	12.75 (0.49)	19.45 (2.22) ^a^

^1^ Lipid adjusted and log-transformed polychlorinated biphenyls (ng/g); ^2^ Geometric means calculated after applying NHANES sampling weights.; ^3^ Age-Adjusted; ^a^ PCB levels were significantly higher in subjects with combined risk factors for PAH (*p* < 0.05).

**Table 4:** Geometric mean of PCB levels above the LOD by age group in subjects with and without combined risk factors for PAH.

	Geometric Mean ^2^ (ng/g) (GSE, *n*)					
	Age: 20–59 Years		Age: 60–74 Years		Age: >75 Years	
**Analyte ^1^**	Control Levels (ng/g) of PCBs	Levels of PCBs in Subjects with Risk Factors for PAH	Control Levels (ng/g) of PCBs	Levels of PCBs in Subjects with Risk Factors for PAH	Control Levels (ng/g) of PCBs	Levels of PCBs in Subjects with Risk Factors for PAH
**PCB74**	6.08 (0.20, 2031)	8.62 (0.72, 80) ^a^	15.15 (0.80, 572)	15.32 (1.19, 125)	22.65 (1.24, 301)	24.75 (2.01, 67)
**PCB99**	5.29 (0.13, 2011)	6.35 (0.38, 76) ^a^	9.43 (0.45, 564)	9.55 (0.78, 123)	11.68 (0.64, 299)	13.15 (1.14, 68)
**PCB118**	7.25 (0.20, 2035)	10.11 (0.85, 80) ^a^	17.51 (1.04, 569)	19.11 (1.93, 125)	24.71 (1.62, 300)	30.54 (2.66, 67)
**PCB138**	19.13 (0.44, 2033)	23.56 (1.65, 80) ^a^	40.17 (1.64, 574)	43.87 (2.42, 124)	50.31 (2.83, 301)	56.73 (3.57, 68)
**PCB153**	27.39 (0.63, 2032)	35.47 (2.55, 80) ^a^	60.88 (2.08, 574)	64.06 (3.49, 125)	76.17 (3.62, 302)	84.56 (5.28, 68)
**PCB180**	12.75 (0.49, 2036)	19.45 (2.22, 79) ^a^	47.01 (1.39, 571)	51.16 (3.22, 126)	59.19 (3.34, 298)	61.65 (5.31, 68)

^1^ Lipid adjusted and log-transformed polychlorinated biphenyls (ng/g). ^2^ Geometric means calculated after applying NHANES sampling weights. ^a^ PCB levels significantly higher in subjects with combined risk factors for PAH (*p* < 0.05).

**Table 5:** Age-adjusted geometric mean PCB levels (ng/g) by race/ethnicity among population in subjects with and without combined risk factors of PAH.

	Geometric Mean ^2^ (ng/g) (GSE, *n*)			
	Control Levels of PCBs		Levels of PCBs in Subjects with Risk Factors for PAH	
Analyte ^1^	Non-Hispanic White	Other	Non-Hispanic White	Other
**PCB74**	8.13 (0.33, 1438)	6.17 (0.17, 1466)	12.86 (0.82, 155) ^a^	12.39 (1.08, 115)
**PCB99**	6.11 (0.19, 1425)	5.81 (0.17, 1449)	7.97 (0.35, 152)	10.46 (1.04, 115) ^b^
**PCB118**	9.11 (0.30, 1438)	8.25 (0.28, 1466)	15.07 (0.95, 154)	16.86 (1.66, 118)
**PCB138**	23.14 (0.59, 1445)	21.14 (0.75, 1463)	32.63 (1.55, 154) ^a^	41.59 (3.56, 118) ^b^
**PCB153**	34.03 (0.85, 1444)	29.52 (1.03, 1464)	49.02 (2.49, 155) ^a^	59.89 (4.89, 118) ^b^
**PCB180**	18.87 (0.79, 1437)	12.69 (0.63, 1468)	33.45 (2.78, 154) ^a^	35.41 (3.53, 119) ^b^

^1^ Lipid adjusted and log-transformed polychlorinated biphenyls (ng/g); ^2^ Geometric means calculated after applying NHANES sampling weights.; ^a^ PCB levels significantly higher in non-Hispanic White with risk factors for PAH vs. non-Hispanic White controls (*p* < 0.05); ^b^ PCB levels significantly higher in race category “other” with combined risk factors for PAH compared to control “other” (*p* < 0.05).

**Table 6:** Serum levels of dioxin-like PCB (ng/g) in the study population, ≥20 years of age, NHANES 1999–2004.

		Mean (ng/g) ^1^ (95% CI)	
Variable	Controls/Subjects with Combined Risk Factors for PAH (*n*)	Control Levels of PCBs	Levels of PCBs in Subjects with Risk Factors for PAH
**Dioxin-like PCBs ^2^**	2915/274	2.81 (2.75–2.87)	3.36 (3.25–3.46) ^a^
**Dioxin-like PCBs_50 ^3^**			
<LOD to 50%	362/22	2.59 (2.57–2.61)	2.62 (2.56–2.68)
≥50%	1379/218	3.52 (3.49–3.56)	3.73 (3.65–3.82)
**Gender**			
Male	1304/182	2.64 (2.56–2.70)	3.16 (3.02–3.29) ^a^
Female	1611/92	2.96 (2.89–3.03)	3.69 (3.55–3.84) ^a^
**Race**			
Non-Hispanic White	1446/155	2.86 (2.79–2.93)	3.35 (3.23–3.48)
Other	1469/119	2.68 (2.62–2.75)	3.38 (3.19–3.57)
**Age**			
20–59	2039/80	2.61 (2.55–2.66)	2.95 (2.79–3.11)
60–74	574/126	3.49 (3.39–3.61)	3.55 (3.37–3.73)
≥75	302/68	3.87 (3.76–3.99)	4.01 (3.86–4.17)
**BMI**			
Normal Weight	985/33	2.78 (2.69–2.86)	3.44 (3.18–3.70) ^a^
Overweight	1031/85	2.79 (2.71–2.86)	3.36 (3.13–3.59) ^a^
Obese	838/139	2.87 (2.79–2.95)	3.29 (3.16–3.42) ^a^
**Income**			
USD 0–24,999	1094/136	2.84 (2.77–2.92)	3.41 (3.23–3.56) ^a^
USD 25,000–54,999	838/69	2.77 (2.69–2.83)	3.33 (3.08–3.57) ^a^
USD 55,000–74,999	275/14	2.72 (2.59–2.84)	3.12 (2.77–3.47)
≥75,000	708/55	2.86 (2.78–2.93)	3.41 (3.15–3.66) ^a^
**Education Level**			
<12th Grade	945/109	2.85 (2.75–2.96)	3.49 (3.27–3.71) ^a^
12th Grade	628/71	2.81 (2.72–2.90)	3.13 (2.92–3.33)
≥12th Grade	1336/94	2.79 (2.72–2.86)	3.44 (3.26–3.62) ^a^
**Smoking**			
Yes	643/44	2.56 (2.49–2.63)	3.08 (2.79–3.36) ^a^
No	746/135	2.96 (2.86–3.07)	3.37 (3.20–3.53) ^a^
**Alcohol use**			
Yes	20/0	2.62 (2.27–2.97)	
No	2892/272	2.81 (2.75–2.87)	3.36 (3.25–3.46) ^a^

^1^ Means calculated after applying NHANES sampling weights.; ^2^ Sum of Dioxin-like PCBs = (74 + 118); Lipid adjusted and log-transformed PCBs.; ^3^ Serum Dioxin-like Levels: <50th percentile vs. ≥50th percentile; ^a^ PCB levels significantly higher in subjects with combined risk factors for PAH (*p* < 0.05).

**Table 7:** Serum levels of non-dioxin-like PCBs (ng/g) in the study population, ≥20 years of age, NHANES 1999–2004.

		Mean (ng/g) ^1^ (95% CI)	
Variable	Controls/Subjects with Combined Risk Factors for PAH (*n*)	Control Levels of PCBs	Levels of PCBs in Subjects with Risk Factors for PAH
**Non-Dioxin-like PCBs ^2^**	2916/275	4.40 (4.36–4.45)	4.88 (4.78–4.98) ^a^
**Non-Dioxin-Like-PCBs 50 ^3^**			
<LOD to 50%	416/33	4.26 (4.24–4.28)	4.29 (4.21–4.36)
≥50%	1404/217	5.12 (5.08–5.15)	5.23 (5.16–5.29)
**Gender**			
Male	1304/183	4.41 (4.35–4.47)	4.83 (4.69–4.97) ^a^
Female	1612/92	4.39 (4.35–4.45)	4.96 (4.84–5.07) ^a^
**Race**			
Non-Hispanic White	1446/155	4.46 (4.40–4.51)	4.84 (4.73–4.95) ^a^
Other	1470/120	4.27 (4.19–4.35)	5.00 (4.85–5.16) ^a^
**Age**			
20–59	2040/80	4.21 (4.16–4.26)	4.48 (4.33–4.62)
60–74	574/127	5.09 (5.02–5.15)	5.14 (5.02–5.25)
≥75	302/68	5.31 (5.22–5.40)	5.41 (5.28–5.53)
**BMI**			
Normal Weight	985/34	4.43 (4.36–4.51)	4.83 (4.58–5.08) ^a^
Overweight	1032/85	4.41 (4.36–4.46)	5.06 (4.89–5.23) ^a^
Obese	838/139	4.34 (4.26–4.42)	4.74 (4.61–4.86) ^a^
**Income**			
USD 0–24,999	1095/136	4.36 (4.29–4.42)	4.94 (4.78–5.09) ^a^
USD 25,000–54,999	838/69	4.37 (4.29–4.44)	4.86 (4.67–5.05) ^a^
USD 55,000–74,999	275/14	4.37 (4.28–4.46)	4.60 (4.27–4.94)
≥75,000	708/56	4.49 (4.44–4.56)	4.92 (4.76–5.09) ^a^
**Education Level**			
<12th Grade	945/109	4.49 (4.39–4.59)	5.08 (4.89–5.27) ^a^
12th Grade	629/71	4.38 (4.29–4.47)	4.69 (4.49–4.90)
≥12th Grade	1336/95	4.38 (4.33–4.43)	4.88 (4.71–5.04) ^a^
**Smoking**			
Yes	643/45	4.31 (4.25–4.38)	4.77 (4.50–5.04) ^a^
No	746/135	4.63 (4.55–4.70)	4.93 (4.78–5.09)
**Alcohol use**			
Yes	20/0	4.24 (3.83–4.64)	
No	2893/273	4.40 (4.36–4.45)	4.88 (4.78–4.98) ^a^

^1^ Means calculated after applying NHANES sampling weights.; ^2^ Sum of Non-Dioxin-like PCBs = (99 + 138 + 153 + 180); Lipid adjusted and log-transformed PCBs.; ^3^ Serum of non-Dioxin-like Levels: <50th percentile vs. ≥50th percentile; ^a^ PCB levels significantly higher in subjects with combined risk factors for PAH (*p* < 0.05).

**Table 8:** Geometric Mean PCB levels (ng/g) among population ≥20 years of age, NHANES 1999–2004.

			Geometric Mean ^2^ (ng/g) (95% CI)	
Analyte ^1^	Controls (*n*)	Subjects with Combined Risk Factors for PAH (*n*)	Control Levels of PCBs	Levels of PCBs in Subjects with Risk Factors for PAH
**PCB74**				
≥LOD	1865	245	11.48 (10.84–12.16)	15.91 (14.45–17.53) ^a^
**PCB99**				
≥LOD	1958	226	8.36 (8.02–8.72)	10.99 (9.96–12.14) ^a^
**PCB118**				
≥LOD	1709	234	15.29 (14.65–15.95)	21.47 (19.13–24.11) ^a^
**PCB138**				
≥LOD	1900	240	33.58 (32.34–34.87)	42.17 (38.56–46.13) ^a^
**PCB153**				
≥LOD	1999	252	46.79 (44.96–48.69)	58.30 (53.75–63.24) ^a^
**PCB180**				
≥LOD	1822	248	37.90 (36.56–39.31)	45.49 (41.36–50.02) ^a^

^1^ Lipid adjusted and log-transformed polychlorinated biphenyls (ng/g); ^2^ Geometric means calculated after applying NHANES sampling weights.; ^a^ PCB levels significantly higher in subjects with combined risk factors for PAH compared to control group (*p* < 0.05).

**Table 9:** Geometric Mean PCB levels (ng/g) by LOD 50%, NHANES 1999–2004.

			Geometric Mean ^2^ (ng/g) (95% CI)	
Analyte ^1^	Controls (*n*)	Subjects with Combined Risk Factors for PAH (*n*)	Control Levels of PCBs	Levels of PCBs in Subjects with Risk Factors for PAH
**PCB074**				
<LOD to 50%	499	22	5.83 (5.74–5.92)	6.27 (5.89–6.68)
≥50%	1366	223	15.17 (14.48–15.89)	17.73 (16.17–19.44)
**PCB099**				
<LOD to 50%	497	26	4.64 (4.59–4.69)	4.68 (4.52–4.84)
≥50%	1461	200	10.53 (10.08–11.00)	12.59 (11.56–13.71)
**PCB118**				
<LOD to 50%	328	23	7.23 (7.12–7.35)	7.43 (7.10–7.78)
≥50%	1381	211	19.01 (18.24–19.82)	24.21 (21.96–26.69)
**PCB138**				
<LOD to 50%	502	26	18.05 (17.79–18.31)	18.89 (17.98–19.83)
≥50%	1398	214	43.59 (41.98–45.27)	48.84 (44.69–53.36)
**PCB153**				
<LOD to 50%	586	33	25.43 (25.02–25.84)	27.06 (25.53–29.23)
≥50%	1413	219	63.33 (61.34–65.38)	69.33 (64.66–74.33)
**PCB180**				
<LOD to 50%	451	30	19.99 (19.69–20.29)	20.13 (18.38–22.05)
≥50%	1371	218	49.33 (47.68–51.04)	53.89 (49.86–58.23)

^1^ Lipid adjusted and log transformed polychlorinated biphenyls (ng/g); ^2^ Geometric means calculated after applying NHANES sampling weights.

**Table 10:** Geometric Mean PCB levels (ng/g) by LOD < 50%, 50–75%, and ≥75%, NHANES 1999–2004.

			Geometric Mean ^2^ (ng/g) (95% CI)	
Analyte ^1^	Controls (*n*)	Subjects with Combined Risk Factors for PAH (*n*)	Control Levels of PCBs	Levels of PCBs in Subjects with Risk Factors for PAH
**PCB074**				
<LOD to 50%	499	22	5.83 (5.74–5.92)	6.27 (5.89–6.82)
50−75%	708	92	10.48 (10.27–10.69)	10.93 (10.21–11.69)
≥75%	658	131	25.57 (24.65–26.53)	27.01 (24.96–29.62) ^a^
**PCB099**				
<LOD to 50%	497	26	4.64 (4.59–4.68)	4.67 (4.52–5.84)
50−75%	758	73	7.07 (6.95–7.19)	7.48 (7.22–7.75)
≥75%	703	127	17.45 (16.57–18.37)	18.43 (16.85–20.15)
**PCB118**				
<LOD to 50%	340	11	7.23 (7.11–7.35)	7.43 (7.10–7.78)
50−75%	758	44	12.52 (12.26–12.79)	13.72 (12.95–14.54) ^a^
≥75%	700	90	34.14 (32.62–35.73)	36.02 (32.43–39.99) ^a^
**PCB138**				
<LOD to 50%	502	26	18.05 (17.79–18.31)	18.89 (17.99–19.83)
50−75%	712	82	30.28 (29.75–30.82)	30.99 (29.39–32.68)
≥75%	686	132	70.46 (67.69–73.34)	72.14 (66.12–78.71) ^a^
**PCB153**				
<LOD to 50%	586	33	25.43 (25.02–25.84)	27.06 (25.05–29.23)
50–75%	713	88	45.42 (44.55–46.30)	47.09 (45.28–48.98)
≥75%	700	131	99.09 (95.46–102.86)	104.39 (96.94–112.41) ^a^
**PCB180**				
<LOD to 50%	451	30	19.99 (19.69–20.29)	20.13 (18.38–22.05)
50–75%	690	90	36.37 (35.58–37.19)	36.73 (34.75–38.82)
≥75%	681	128	75.99 (73.65–78.40)	81.67 (77.15–86.44) ^a^

^1^ Lipid adjusted and log-transformed polychlorinated biphenyls (ng/g); ^2^ Geometric means calculated after applying NHANES sampling weights.; ^a^ PCB levels significantly higher in subjects with combined risk factors for PAH compared to control group (*p* < 0.05).

**Table 11:** Estimated ORs (95% CI) for PCB congener levels in participants with the combined risk factors for PAH compared to control by LOD ≥ 50%.

Analyte ^1^	Controls (*n*)	Subjects with Combined Risk Factors for PAH (*n*)	Unadjusted OR (95% CI)	Adjusted OR ^2^ (95% CI)	Adjusted OR ^3^ (95% CI)	Adjusted OR ^4^ (95% CI)
**PCB74**						
≥50%	1406	223	3.38 (3.37–3.40)	4.00 (3.98–4.01)	1.95 (1.94–1.96)	2.31 (2.30–2.32) ^a^
**PCB99**						
≥50%	1845	200	2.32 (2.31–2.32)	2.38 (2.37–2.39)	1.44 (1.43–1.44)	1.50 (1.49–1.50) ^a^
**PCB118**						
≥50%	1411	211	2.54 (2.53–2.55)	2.83 (2.82–2.84)	1.51 (1.50–1.51)	1.67 (1.66–1.68) ^a^
**PCB138**						
≥50%	1837	214	2.19 (2.18–2.20)	2.22 (2.22–2.23)	1.27 (1.27–1.28)	1.28 (1.27–1.28) ^a^
**PCB153**						
≥50%	1835	219	2.20 (2.19–2.21)	2.18 (2.17–2.19)	1.38 (1.380–1.389)	1.37 (1.36–1.37) ^a^
**PCB180**						
≥50%	1832	218	1.98 (1.97–1.99)	1.95 (1.95–1.96)	1.37 (1.37–1.38)	1.30 (1.29–1.30) ^a^

^1^ Lipid adjusted and log-transformed polychlorinated biphenyls (ng/g); ^2^ Adjusted for gender; ^3^ Adjusted for age, race, and BMI. ^4^ Adjusted for age, race, BMI, gender; ^a^ *p* < 0.05.

**Table 12:** Estimated ORs (95% CI) for PCB congener levels in subjects with the combined risk factors for PAH compared to control by LOD 50th to 75th percentile and ≥75th percentile.

Analyte ^1^	Controls (*n*)	Subjects with Combined Risk Factors for PAH (*n*)	Unadjusted OR (95% CI)	Adjusted OR ^2^ (95% CI)	Adjusted OR ^3^ (95% CI)	Adjusted OR ^4^ (95% CI)
**PCB074**						
50−75%	725	92	2.70 (2.69–2.71)	3.02 (3.01–3.04)	1.94 (1.93–1.95)	2.18 (2.17–2.19) ^a^
≥75%	681	131	4.32 (4.31–4.34)	5.82 (5.79–5.84)	1.98 (1.78–1.99)	2.65 (2.63–2.66) ^a^
**PCB099**						
50−75%	953	73	1.77 (1.76–1.77)	1.78 (1.77–1.78)	1.38 (1.37–1.38)	1.40 (1.40–1.41) ^a^
≥75%	892	127	3.00 (2.99–3.01)	3.18 (3.17–3.19)	1.52 (1.51–1.52)	1.62 (1.61–1.63) ^a^
**PCB118**						
50−75%	737	76	1.81 (1.80–1.82)	1.93 (1.92–1.94)	1.34 (1.34–1.36)	1.43 (1.42–1.44) ^a^
≥75%	674	135	3.55 (3.53–3.57)	4.37 (4.35–4.39)	2.55 (2.54–2.56)	2.20 (2.56–2.59) ^a^
**PCB138**						
50−75%	941	82	1.82 (1.81–1.83)	1.84 (1.83–1.85)	1.21 (1.21–1.22)	1.22 (1.21–1.23) ^a^
≥75%	896	132	2.67 (2.66–2.68)	2.74 (2.73–2.75)	1.37 (1.37–1.38)	1.37 (1.36–1.38) ^a^
**PCB153**						
50−75%	938	88	1.98 (1.97–1.99)	1.99 (1.99–2.00)	1.42 (1.41–1.43)	1.43 (1.42–1.44) ^a^
≥75%	897	131	2.46 (2.45–2.47)	2.49 (2.48–2.50)	1.30 (1.30–1.31)	1.26 (1.25–1.27) ^a^
**PCB180**						
50−75%	935	90	1.75 (1.75–1.76)	1.75 (1.74–1.76)	1.31 (1.31–1.32)	1.29 (1.28–1.29) ^a^
≥75%	897	128	2.32 (2.31–2.33)	2.26 (2.25–2.27)	1.51 (1.50–1.52)	1.33 (1.32–1.34) ^a^

^1^ Lipid adjusted and log-transformed polychlorinated biphenyls (ng/g); ^2^ Adjusted for gender; ^3^ Adjusted for age, race, and BMI. ^4^ Adjusted for age, race, BMI, gender; ^a^ *p* < 0.05.

**Table 13:** Estimated ORs (95% CI) for dioxin-like and non-dioxin-like PCB levels in subjects with the combined risk factors for PAH compared to control by LOD ≥ 50th and ≥75th percentile.

	Controls (*n*)	Subjects with Combined Risk Factors for PAH (*n*)	Unadjusted OR (95% CI)	Adjusted OR ^1^ (95% CI)	Adjusted OR ^2^ (95% CI)	Adjusted OR ^3^ (95% CI)
**Dioxin_Like_PCBs_50 ^5,6^**						
**≥50%**	1415	218	2.64 (2.63–2.66)	2.95 (2.93–2.96)	1.53 (1.52–1.54)	1.73 (1.72–1.74) ^a^
**Dioxin_Like_PCBs_LOD_to75 ^4,7^**						
**≥75%**	1112	101	2.51 (2.51–2.52)	2.95 (2.95–2.97)	1.51 (1.51–1.52)	1.75 (1.75–1.76) ^a^
**Non_Dioxin_Like_PCBs_50 ^5,6^**						
**≥50%**	1841	217	1.58 (1.79–1.59)	1.59 (1.58–1.60)	1.00 (1.00–1.01)	0.99 (0.99–0.99)
**Non_Dioxin_Like_PCBs_LOD_to75 ^5,7^**						
**≥75%**	1487	118	1.51 (1.50–1.51)	1.52 (1.52–1.53)	1.00 (0.99–1.00)	0.51 (0.95–0.96)

^1^ Adjusted for gender; ^2^ Adjusted for age, race, and BMI; ^3^ Adjusted for age, race, BMI, gender; ^4^ Dioxin-like PCBs: Sum of lipid adjusted and log transformed PCB Congeners (74 + 118); ^5^ Non-Dioxin-like PCBs: Sum of lipid adjusted and log transformed PCB Congeners (99 + 138 + 153 + 180); ^6^ Serum PCB levels < 50th percentile vs. ≥50th percentile.; ^7^ Serum PCB levels < 75th percentile vs. ≥75th percentile. ^a^ *p* < 0.05.

## Data Availability

The data presented in this study are available on request from the corresponding author.
